# Surfactant protein A: a candidate predictive biomarker of mortality in acute respiratory distress syndrome in sepsis

**DOI:** 10.1186/cc13407

**Published:** 2014-03-17

**Authors:** V Moroz, A Kuzovlev, A Goloubev

**Affiliations:** 1 V.A. Negovsky Scientific Research Insitute of General Reanimatology, RAMS, Moscow, Russia

## Introduction

Prediction of mortality in septic acute respiratory distress syndrome (ARDS) is a problem of great significance. The aim of this study was to investigate the role of surfactant protein A (SPA) as a candidate predictive biomarker of mortality in ARDS.

## Methods

The observational study in ICU ventilated septic patients with peritonitis (65%), pancreonecrosis (20%) and mediastinitis (15%) was performed in 2010 and 2013. ARDS was diagnosed and staged according to the V.A. Negovsky Research Institute criteria [1,2] and the Berlin definition. Plasma SPA was measured on ARDS diagnosis (day 0) and days 3 and 5 by the immunoenzyme essay (BioVendor, USA). Patients were treated according to the international guidelines. Data were statistically analyzed by STATISTICA 7.0, ANOVA method, and presented as median and 25th to 75th percentiles, ng/ml; *P *< 0.05 was considered statistically significant. Areas under the receiver operating curves were calculated.

## Results

Eighty-five patients (out of 300 screened) were enrolled in the study according to the inclusion/exclusion criteria. Patients were assigned into groups: ARDS (*n *= 50, 48 ± 5.7 years old, M/F 35/15, mortality 26%) and noARDS (*n *= 35, 46 ± 8.7 years old, M/F 30/5, mortality 23%). Groups were comparable in APACHE II scores. In the ARDS group SPA was higher at all points than in the noARDS group (day 0: 32.6, 25 to 75 IQR 18.2 to 60.7 vs. 23.4, 25 to 75 IQR 17.8 to 28.6; day 3: 31.5, 25 to 75 IQR 20.9 to 31.5 vs. 24.6, 25 to 75 IQR 19.7 to 24.6; day 5: 32.5, 25 to 75 IQR 17.3 to 66.4 vs. 22.5, 25 to 75 IQR 13.4 to 29.5, *P *< 0.05). The plasma SPA was significantly lower in surviving versus dead patients with ARDS (day 0: 20.9, 25 to 75 IQR 13.0 to 35.7 vs. 45.7,25 to 75 IQR 23.5 to 67.9; day 3: 25.5, 25 to 75 IQR 11.8 to 35.5 vs. 45.0, 25 to 75 IQR 29.6 to 68.4; day 5: 24.5, 25 to 75 IQR 11.4 to 33.6 vs. 49.6, 25 to 75 IQR 31.3 to 79.0, *P *< 0.05). Plasma SPA on day 0 had a good capacity for prediction of mortality in ARDS patients: SPA on day 0 ≥38.8 ng/ml yielded a sensitivity of 65% and specificity of 80% (aUc 0.74; 95% Cl 0.577 to 0.866; *P *= 0.0026).

## Conclusion

Plasma SPA level ≥38.8 ng/ml on the day of ARDS diagnosis is a sensitive and specific candidate biomarker of mortality prediction in ARDS septic patients.
